# Public Education on Epistaxis Management: Does a Video Help?

**DOI:** 10.7759/cureus.106436

**Published:** 2026-04-04

**Authors:** Joshua M Sorrentino, Lauren A DiNardo, Abraham A Kassem, Alyssa Reese, Douglas P Nanu, Ryan Nagy, Michele M Carr

**Affiliations:** 1 Otolaryngology, Jacobs School of Medicine and Biomedical Sciences, University at Buffalo, Buffalo, USA; 2 Otolaryngology, University of Rochester Medical Center, Rochester, USA; 3 Medicine, Jacobs School of Medicine and Biomedical Sciences, University at Buffalo, Buffalo, USA; 4 Medicine, Washington State University, Spokane, USA; 5 Emergency Medicine, Memorial Health System, Marietta, USA

**Keywords:** educational video, epistaxis, first aid, health literacy, patient education

## Abstract

Introduction

Epistaxis is a common complaint seen by otolaryngologists and one of the most common otolaryngological emergencies. The aim of our study is to identify educational gaps in home epistaxis management and determine the efficacy of an educational video in increasing layperson knowledge of epistaxis treatment.

Methods

Adults aged 18 years and older were recruited in pediatric otolaryngology clinics and online. The survey consisted of pre- and post-test questions, with a locally produced educational video on epistaxis management presented between assessments. Demographics collected included age, gender, race, and parental status. Experience with nosebleeds and nasal cautery was queried. The assessment included 10 multiple-choice and true/false questions regarding active nosebleed management and prevention techniques.

Results

A total of 145 participants completed both assessments. The majority were female (N = 87, 60.0%), younger than 30 years (N = 120, 82.8%), and of Caucasian/White race (N = 103, 71.0%). Nineteen participants (13.1%) identified as parents, and 109 (75.2%) reported personal or child experience with epistaxis. Post-test scores were significantly higher than pre-test scores (mean difference = 2.21, t(144) = 13.10, p < 0.001). Parents had significantly higher post-test scores compared to non-parents (p = 0.025). There was no statistically significant difference in post-test scores based on gender, age, race, history of epistaxis, or history of nasal cautery.

Conclusion

The educational video increased layperson knowledge about epistaxis. Future research is needed to understand how to best use educational videos in the clinical setting.

## Introduction

Epistaxis is a common complaint and one of the most frequent otolaryngologic emergencies [1]. Approximately 60% of individuals experience at least one episode during their lifetime, and about 10% of these require medical intervention [1,2]. Notably, an estimated 6% of those with epistaxis ultimately seek medical attention, underscoring its burden on healthcare systems [3]. While many cases are idiopathic, epistaxis may also indicate a more serious underlying pathology. Accordingly, a thorough history is essential to guide long-term management [4]. Because epistaxis frequently occurs outside the hospital setting, it is critical for the public to understand how to manage it acutely [5]. However, studies demonstrate a widespread lack of knowledge regarding basic first-aid techniques. In a survey by Saleem et al., 62.6% (N = 1,760) of participants answered two or fewer of six questions correctly on epistaxis first aid, which assessed site and duration of pressure, head positioning, use of ice, and nasal packing [5]. Similarly, only 30% of caregivers correctly performed first-aid management for their child prior to seeing an otolaryngologist [6]. Educating the general population on how to manage nosebleeds may improve the quality of life of those who are affected by this condition [6]. A recent survey found that 58% of adults identified social media as the best source to increase awareness on epistaxis management [5]. However, the information on these platforms is often unreliable or inconsistent. With more adults turning to social media platforms like YouTube (Google LLC, Mountain View, CA, USA) for medical information on epistaxis management, it is essential to evaluate the quality of available content and consider how otolaryngologists can contribute accurate, accessible educational resources [7,8]. Despite this trend, limited research has explored what adults know about managing epistaxis. The goal of this study was to identify existing knowledge gaps, provide targeted education via a brief instructional video, and evaluate its effectiveness in improving lay understanding of epistaxis management.

## Materials and methods

Study design and participants

This was a prospective pre-post interventional survey study designed to evaluate whether a brief educational video improved layperson knowledge of epistaxis management. The study was approved by the State University of New York (SUNY) at Buffalo Institutional Review Board (IRB #00005886). All participants provided informed consent prior to participation.

Data were collected between January and October 2022. Adults aged 18 years or older were eligible to participate. Participants were recruited in person from a pediatric otolaryngology clinic and electronically through online platforms, including WhatsApp (living in Portland group) (Meta Platforms, Inc., Menlo Park, CA, USA), Facebook (Meta Platforms, Inc.), SurveyCircle (www.surveycircle.com), and email distribution lists. Participation was voluntary and anonymous. Only individuals who completed both the pre-test and post-test assessments were included in the final analysis.

Survey instrument and educational intervention

The survey was created using Google Forms (Google LLC), a freely available web-based platform. The survey instrument was developed locally by the study team and was not derived from a copyrighted or proprietary scoring system.

The assessment consisted of 10 multiple-choice and true/false questions designed to evaluate knowledge of acute epistaxis management and prevention strategies. Questions were developed based on established first-aid recommendations for anterior epistaxis management [3,9]. Content domains included appropriate head positioning, correct location and duration of nasal compression, use of topical vasoconstrictors, indications for seeking medical evaluation, and preventative strategies.

Participants first completed a pre-test assessing baseline knowledge. They were then shown a locally produced educational video (https://www.youtube.com/watch?v=1SLe1D5NtAQ) explaining recommended first-aid management of epistaxis. Immediately after viewing the video, participants completed the same 10-question assessment as a post-test. Total scores ranged from 0 to 10, with higher scores indicating greater knowledge.

Variables and outcome measures

Participants self-reported demographic information including age, gender, race/ethnicity, and parental status. They were also asked whether they or their child had previously experienced epistaxis and whether they or their child had undergone nasal cautery.

The primary outcome measure was the change in total correct responses between pre-test and post-test scores. Secondary analyses evaluated associations between post-test scores and demographic characteristics and prior experience with epistaxis.

Statistical analysis

Statistical analyses were performed using Stata version 15 (StataCorp, College Station, TX, USA), licensed through our institution. Continuous variables are reported as means with standard deviations (SDs), and categorical variables are reported as frequencies and percentages.

Differences between pre-test and post-test scores were evaluated using a paired t-test. Comparisons of post-test scores between independent groups (e.g., gender, parental status, history of epistaxis, and history of nasal cautery) were conducted using the Mann-Whitney U test. Test statistics (t- and U-values) are reported in the Results and corresponding tables. Statistical significance was defined as p < 0.05. No proprietary classification systems, staging criteria, or licensed scoring instruments were used in this study.

## Results

A total of 145 participants completed both the pre-test and post-test assessments and were included in the final analysis. Demographic characteristics are summarized in Table 1. The majority of participants were female (N = 87, 60.0%), younger than 30 years of age (N = 120, 82.8%), and of Caucasian/White race (N = 103, 71.0%). Nineteen participants (13.1%) identified as parents.

**Table 1:** Demographics of 145 participants.

Demographics	N (%)
Gender	
Female	87 (60.0)
Male	53 (36.6)
Non-binary	2 (1.4)
Other/prefer not to say	3 (2.1)
Race	
Caucasian/White	103 (71.0)
African American/Black	5 (3.4)
Asian	12 (8.3)
Hispanic	9 (6.2)
Native American	3 (2.1)
Multiracial	10 (6.9)
Prefer not to say	3 (2.1)
Age	
Less than 30 years old	120 (82.8)
30-45 years old	19 (13.1)
45 years or older	6 (4.1)
Parent	
Yes	19 (13.1)
No	126 (86.9)

A total of 109 participants (75.2%) reported a personal or child history of epistaxis, and 13 (9.0%) reported a history of nasal cautery (Table 2). Post-test scores did not significantly differ based on personal or child history of epistaxis (Mann-Whitney U = 2,114, p = 0.479) or history of nasal cautery (U = 834, p = 0.866).

**Table 2:** Parent status and history of epistaxis or nasal cautery and difference of total correct responses. Values compared using the Mann-Whitney U test.

Variable	Mean post-test score	N (%)	U	p-value
Personal or child history of epistaxis (yes)	6.82	109 (75.2)	2,114	0.479
Personal or child history of epistaxis (no)	6.47	36 (24.8)		
Personal or child history of nasal cautery (yes)	6.62	13 (9.0)	834	0.866
Personal or child history of nasal cautery (no)	6.74	132 (91.0)		

Differences in post-test scores by gender and parental status are presented in Table 3. There was no statistically significant difference in post-test scores between females and males (U = 2,717, p = 0.072). However, participants who identified as parents had significantly higher post-test scores compared to non-parents (U = 1,574, p = 0.025).

**Table 3:** Mean post-test score: differences by gender and parent status. Values compared using the Mann-Whitney U test.

Variable	Mean post-test	Standard deviation	U	p-value
Gender				
Female	7.0	1.8	2,717	0.072
Male	6.5	1.7		
Non-binary	4.7	0.6		
Prefer not to say	6.7	1.8		
Parent				
Yes	7.6	1.2	1,574	0.025
No	6.6	1.9		

Post-test scores were significantly higher than pre-test scores. The mean pre-test score was 4.5 (SD = 1.8), and the mean post-test score was 6.7 (SD = 1.8), representing a mean increase of 2.21 points (t(144) = 13.10, p < 0.001).

## Discussion

Our findings suggest that a brief educational video could be used to improve layperson knowledge of epistaxis management. Prior studies have consistently found a lack of epistaxis management knowledge in the public. Strachan et al. found that among 500 participants, only 35% correctly identified where to pinch during an active bleed, 36% knew that the head should be tilted forward during an active bleed, and just 11% answered both of those statements correctly [10]. Similarly, Al-Shehri et al. found that only 58.3% of adults recognized tilting the head forward as the correct position during epistaxis [7]. Saleem et al. surveyed 1,760 adults and found even lower awareness: only 11.3% believed pressure could stop a nosebleed, 5.6% knew to pinch the lower nose, and 4.8% knew to tilt the head forward [5]. Consistent with these studies, our cohort demonstrated limited baseline knowledge, with a mean pre-test score of 45%.

Given that 15% of emergency department visits for epistaxis are resolved with simple pressure or topical vasoconstrictors like oxymetazoline, proper at-home management could reduce unnecessary healthcare utilization [9]. These interventions are low-risk, require no specialized equipment, and can be performed at home within minutes, yet they remain underutilized due to limited public awareness.

With epistaxis having a bimodal distribution, most common in children under age 10 and adults over age 50, it is critical for adults to be equipped with basic first aid for epistaxis [11]. A prior study on pediatric epistaxis found that 80.1% of parents recognized that the initial step in management is to sit the child upright and apply pressure [12]. In our study, adults who identified as parents answered significantly more questions correctly. These findings, consistent with prior research, suggest that parents may have greater awareness of acute epistaxis management, likely due to increased exposure and experience managing epistaxis in their children.

The public may seek information on epistaxis management from a variety of sources; however, knowledge gaps exist even among healthcare professionals. Saleem et al. reported that 25.1% of participants identified doctors and 13.1% identified nurses or other healthcare workers as the best sources for increasing epistaxis awareness [5]. Yet, in practice, accuracy among providers is limited: only 43% of emergency medicine physicians, 24% of residents rotating in the emergency department, 19% of family medicine physicians, and 8% of emergency department nurses knew the correct location to apply pressure to control acute epistaxis, despite high self-reported confidence levels [13]. Seventy-five percent of emergency medicine physicians were “very confident” in their incorrect response on compression location [13]. This is further emphasized by Lavy and Koay, who found that only about 60% of patients previously treated for epistaxis by their primary care provider or emergency medical staff recalled being taught any first aid advice techniques, compared to 100% who were seen by otolaryngologists [14]. Thus, adults in the general population may also be receiving incorrect information from professionals they trust.

Educational videos have demonstrated utility in other contexts for patients and parents. Cassady et al. reported that after watching a pre-operative video, parents had an immediate significant increase in knowledge of pediatric anesthesia [15]. Similarly, another study found a significant increase in parental knowledge of post-operative pain in their child after an educational video and questions [16].

As more adults turn toward social media for medical information, it is important to consider the current quality of that information. For epistaxis management, information on YouTube is highly variable. Haymes and Harries reviewed 50 YouTube videos and found that most included only two of eight recommended epistaxis management criteria [17]. These findings highlight the need for otolaryngologists to develop educational YouTube content to disseminate accurate medical knowledge to both the public and medical professionals in other specialties.

Limitations

The primary limitation of our study is the small sample size. As a convenience sample, the population was not evenly distributed across demographic categories. The study was not designed to have a separate control group, as each participant served as their own control with a pre/post design. Demographic information was limited, and education level and healthcare worker status were not included. Further research should include a larger, more diverse sample of the general population across the country to better understand what adults know about epistaxis management. Also, the assessment was based on knowledge emphasized in our institutional video and was not validated externally. Other institutions may emphasize other aspects of epistaxis management. Finally, although improvements in scores were statistically significant, the clinical significance remains uncertain. Further studies should examine whether these gains translate into sustained knowledge retention or actual behavior change.

## Conclusions

Watching a brief educational video was associated with increased layperson knowledge of epistaxis management. The next step is to determine how educational videos can best be integrated into clinical practice. Future research should investigate broader applications of video-based education for other common first-aid topics with demonstrated gaps in public knowledge.
